# The relevance of the cross-wavelet transform in the analysis of human interaction – a tutorial

**DOI:** 10.3389/fpsyg.2014.01566

**Published:** 2015-01-09

**Authors:** Johann Issartel, Thomas Bardainne, Philippe Gaillot, Ludovic Marin

**Affiliations:** ^1^Multisensory Motor Learning Laboratory, School of Health and Human Performance, Dublin City UniversityDublin, Ireland; ^2^Geophysics Imagery Laboratory, Université de Pau et des Pays de l’AdourPau, France; ^3^ExxonMobil Upstream Research Company, Hydrocarbon Systems Division, Structure, Petrophysics & GeomechanicsHouston, TX, USA; ^4^Movement to Health Laboratory, Sciences et Techniques des Activités Physiques et Sportives, EuroMov, University Montpellier 1Montpellier, France

**Keywords:** cross-wavelet transform, relative phase, human interaction, plurifrequential time-series, joint action

## Abstract

This article sheds light on a quantitative method allowing psychologists and behavioral scientists to take into account the specific characteristics emerging from the interaction between two sets of data in general and two individuals in particular. The current article outlines the practical elements of the cross-wavelet transform (CWT) method, highlighting WHY such a method is important in the analysis of time-series in psychology. The idea is (1) to bridge the gap between physical measurements classically used in physiology – neuroscience and psychology; (2) and demonstrates how the CWT method can be applied in psychology. One of the aims is to answer three important questions WHO could use this method in psychology, WHEN it is appropriate to use it (suitable type of time-series) and HOW to use it. Throughout these explanations, an example with simulated data is used. Finally, data from real life application are analyzed. This data corresponds to a rating task where the participants had to rate in real time the emotional expression of a person. The objectives of this practical example are (i) to point out how to manipulate the properties of the CWT method on real data, (ii) to show how to extract meaningful information from the results, and (iii) to provide a new way to analyze psychological attributes.

## INTRODUCTION

In psychology, numerous studies investigate the interactions between different components of a living system or between different living systems. The idea of interaction is meant here as the expression of action that occurs as two or more ‘objects’ have an effect upon one another (Sebanz and Knoblich, 2009). Numerous studies in psychology also investigate the progression in time of these interactions (Boyd et al., 2011). The data set obtained when analyzing human interaction, and even more when analyzing the temporal evolution of human interaction, contains a lot of powerful information, which is sometimes difficult to quantify and interpret. The cross-wavelet^1^ transform (CWT) method offers a way to overcome such difficulties. The ‘cross’ WT method is a technique that characterizes the interaction between the wavelet transform (WT) of two individual time-series. Even if the purpose of this tutorial is essentially based on a description of the interaction between time-series (i.e., the CWT method), some explanations will refer to the WT method when the methodological explanations are not focused on the interaction between time-series.

The core of this tutorial will be mainly based on practical explanations rather than reviewing, once more, all mathematics behind those techniques. Readers interested by mathematical details can refer to “A practical guide on time-frequency analysis to study human motor behavior: the contribution of WT” by Issartel et al. (2006) or they can refer to a tutorial in neuroscience from Samar et al. (1999), or in psychology from Ihlen and Vereijken (2010). Various application of the WT can be found in psychophysiology (Frantzidis et al., 2014), in the analysis of event-related potentials for Schizophrenic patients (Roth et al., 2007) and behavioral neuroscience (Inoue and Sakaguchi, 2014; Schmidt et al., 2014). The illustrations will be based on both synthetic and real data sets. Step by step, we aim to demonstrate the advantages of the CWT in psychology for non-experts in signal processing research. Our purpose is to describe and illustrate the relevance of the CWT method (and the associated tools) to study the interaction phenomenon between different components of a living system or between different living systems. By the end the article the reader should have an understanding of the strength and advantage of the CWT method and be able to evaluate the benefits of this method on his/her experimental signals. The mathematical aspects will not be detailed in this tutorial. Such a description would be the topic of another article. We deliberately kept our focus on how to understand the potential use of the CWT method and on how to extract the useful information when analyzing time-series in psychology.

These interaction phenomena are permanently present in our everyday life. Such ubiquity implies that all psychological fields are involved in the study of interaction phenomena and most of them tend to use time-series analyses. For instance, in social psychology Gernigon et al. (2004) demonstrate that in sport, states of involvement toward mastery, performance-approach, and performance-avoidance goals flow, are temporally interrelated. Clinical studies also examine the interaction phenomenon. For instance, Slewa-Younan et al. (2004) illustrated the difference in brain functional connectivity between males and females with schizophrenia. In the wide field of behavioral studies, one can find numerous cases of research on interaction phenomena using time-series analyses. For example, in human communication listeners mirror the movements of a storyteller (Bavelas et al., 1988; Voutilainen et al., 2014), in music, body movement (e.g., foot tapping) is coordinated with the melody (Jones, 1993), and also in human conversational interaction (Boker and Rotondo, 2002; Guastello et al., 2005; Pincus and Guastello, 2005; Kupetz, 2014), in the development of infant joint attention and social cognition (Mundy and Jarrold, 2010), in collective behavior organization like rapturous applause (Neda et al., 2000), or even in handwriting when a synchronization phenomenon appears between the rhythmic movement of the fingers and the wrist (Sallagoïty et al., 2004). The above list of studies, focusing on the interaction phenomena, is obviously not exhaustive. Other examples will be presented, in this article, while introducing the core components of the CWT method.

The concept of interaction refers to a synchronization phenomenon (Kelso, 1995, for a review) between the elements that compose a system or between different systems (Moraru et al., 2005). Moraru et al. (2005) defined the general synchronization phenomenon as the characterization and quantification of interdependencies between different system components. The term synchronization is a construct used in numerous fields to denote the temporal relationship between events. This relationship occurs in a variety of contexts, for example, when sport fans start a wave across a stadium, when two people emotionally “click” with one another (e.g., parent–infant synchrony – Granic and Lamey, 2002), or the sleep–wake rhythm with the light–dark cycle. There is not only synchronization when two components are perfectly coordinated (such as two soldiers in lockstep – i.e., in phase with each other), but as long as any kind of interaction exists between two (or more) components. In such a context, although it seems paradoxical, two ex-partners opposed while divorcing, synchronize their actions. When partner 1 makes a decision, partner 2 frequently contradicts partner 1. In this example, they both “move” in the same time but in the opposite direction (anti-phase). This synchronization phenomenon can also be observed, on a daily basis, when friends mimic each other posture and even speech patterns while sipping coffee. In this context, synchronization occurs with certain latency that is also a common characteristic when systems are interacting with each other.

The CWT method allows us (i) to measure the degree of synchronization phenomenon between different components and conjointly (ii) the evolution over time of the studied phenomenon (i.e., times-series data set). For example, it allows the extraction of information about how a behavioral state changes, how long it takes to progress from one state to another and how many states occur in a given time period. In addition, similarity/differences between two time-series (e.g., the similarity between two people’s behavioral states) can be identified. If these behavioral states do not have a similar dynamic (i.e., different temporal evolution), the CWT method helps quantifying how different they are, and what is the time lag between the two different behavioral states? In short, this method is able to yield information on the spatio-temporal organization between two time-series, or in other words how two time-series evolve one against the other. We will address different examples based either on synthetic or real data sets to demonstrate how this method can be helpful to interpret time-series.

This tutorial is organized in an accessible way for readers with different backgrounds. First of all, we demonstrate WHAT the WT is and how it is related to the notions of synchronization and relative phase (RP). Secondly, we explain WHY the analyses performed with this method could offer a beneficial way to interpret time-series data obtained from psychological experiments. In the third section, we subsequently illustrate WHO could use this method. In other words, which areas in psychology could potentially benefit from using this method. The fourth section is dedicated to an explanation of the time-series specificities required to use this method (called the “WHEN” section). We explore the types of time-series that could be used and those that seem to present some limitations. Then, we detail the WT method using a synthetic example to illustrate HOW this method can be applied on time-series data set and HOW to extract useful information. Throughout this section, we associate a synthetic example with a psychologically concrete related example to contextualize this method for the reader. Finally, a real set of data is analyzed. The data has been taken from an experiment assessing the dynamics of emotional state. The idea is to analyze this real time-series from the extraction of the variables up to the interpretation phase of the results.

## WHAT IS THE CROSS-WAVELET TRANSFORM METHOD?

As stated in introduction, the notion of synchronization plays a key role in the understanding of the CWT method. First of all, we will define the concept of synchronization. We will demonstrate how the synchronization is related to two other elements, namely the RP and the frequency. These explanations will constitute the fundamental knowledge needed to understand the CWT method.

### SYNCHRONIZATION

As described earlier on, the synchronization phenomenon can be considered as an ongoing relationship between different structures or systems. The systematic study of this experimental as well as theoretical phenomenon has been widely investigated in physics since Appleton (1922) and van der Pol (1927; for a review, Pikovsky et al., 2003), but was only introduced in experimental psychology by Kelso (1981) in the theoretical framework of coupled dynamical systems. This dynamical system can be applied to understanding appraisal or emotion for example. Nowadays many scientists will consider emotion as non-linear, dynamic, distributed where self-organized patterns emerge from the interaction between the components of a system. This spontaneous emergence of order, coherence and synchronization have been examined and analyzed with different methods (e.g., the windowed cross-correlation function, Boker et al., 2002). Although, there are multiple ways to observe the correlation or the coherency between two components, only one variable can describe and precisely measure the phase synchrony between different components. The phase synchrony, also called the RP, is a variable commonly applied to psychology because it conveys the essential aspects of a behavior by summarizing the relations between the components of a system. It examines the relationships between spatial and temporal information.

### RELATIVE PHASE

Traditionally, measures of RP have been used to quantify the coordination between two or more components during an activity (see Kelso, 1995, for a review) as well as to determine the transition from one behavior to another either intentionally (Scholz and Kelso, 1990) or unintentionally (Kelso, 1981) such as two gaits unintentionally synchronized when two partners are walking in the street side by side. The RP can be understood as a relational variable that measured the ordering of the interaction among components. The self-organized interaction between components leads to the emergence of a behavioral dynamics. In other words, the RP capture the coordination between the components. A coordination where the components are moving in the same direction, at the same time, have a RP of 0° (also called in-phase) while a coordination with a phase shift of 180° is called anti-phase.

For instance, Kelso (1981) analyzed the coordination/coupling between two fingers and quantified phase relationships as the difference between successive flexions or extensions in the oscillations of the fingers. This approach is called the point estimate of the RP (see Von Holst, 1973) and corresponds to a discrete relative phase (DRP). The DRP computes the RP between two time-series as the temporal difference between two inflection points in the time-series. For example, in a dynamic measurement of psychological states, like states of goal involvement toward mastery and performance-avoidance goals flow (Gernigon et al., 2004), it is possible to measure a reference point each time the states are changing direction (i.e., the inflection point between the end of the increasing state of involvement toward mastery and the beginning of the decreasing of this state). Psychological momentum is considered as a dynamical phenomenon defined as “a positive or negative dynamics of cognitive, affective, motivational, physiological, and behavioral responses (and their couplings) to the perception of movement toward or away from either an appetitive or aversive outcome” (Gernigon et al., 2010, p. 397). In this citation, the key words – positive/negative or appetitive/aversive – highlight to the notion of continuous dynamical changes occurring in the coupling between components. The interactions between cognitive vs. physiological or affective vs. motivational components, in a given situation, are on constant evolution (up and down, slow or fast, etc …). In the study by Gernigon et al. (2010), they manipulated the scenarios of performance in a table tennis match by increasing or decreasing the scores gaps between players. They assessed the anxiety or self-confidence levels of a participant observing the video of a match. They were interested in understanding how the perception of moving toward or away from the desired outcome (winning) affect the above mentioned psychological determinants. They measured these determinants after each point during the match. This discrete measurement provides a snapshot of these determinants in a sequential basis (point-to-point) but does not take into account how determinants such as expectations of success, anxiety, perceived situational threat evolve from moment-to-moment^2^. The RP between two of these psychological determinants would capture the behavioral dynamics between these components. In such a case, the RP could provide, for example, an expression of the spatio-temporal organization between two goal involvement states. This DRP method can be applied to a time-series with changes in frequency (e.g., slow increases vs. fast decreases of self-confidence) and amplitude (minor vs. major changes) and allows us to describe the evolution of the RP as a function of time. However, the DRP only computes one or two measures of RP per cycle (i.e., time between two inflection points). Therefore, the DRP does not provide a continuous estimation of the variability of the RP, or an accurate estimation of the time of occurrence of a transition (i.e., when the behavior switches from one state to another). This is crucial information while studying participants’ behavioral modifications. To remedy these limitations, another method of computing the continuous relative phase (CRP) between two time-series has been developed (Kelso, 1995).

While the DRP gives one or two values of RP per cycle, the CRP provides the RP for every point of the time-series. This higher resolution gives a more accurate picture of phase transition, for example, the moment of transition from one behavioral state to another can be quantified (see Kelso et al., 1987). One of the most famous methods used to analyze the continuous RP is called the Hilbert transform, which provides higher resolution results than the DRP methods (Fuchs et al., 1996; Rosenblum et al., 1996, 2001). This method gives both the instantaneous phase and amplitude of the time-series, which is an advantage because with one method, it is possible to measure both the amplitude and the RP of time-series. Mathematically speaking, this method can be used for non-harmonic and non-stationary time-series. For these reasons, this methodology has been successfully applied in behavioral psychology in order to detect phase synchronization between two non-stationary time-series corresponding, for instance, to the displacement of two tennis players (Palut and Zanone, 2004) or to get an accurate measure of the time of intentional switches from one graphic pattern to another in a handwriting task (Sallagoïty et al., 2004).

So far, the Hilbert transform seems to be a relevant method to detect the phase synchronization between two time-series providing that the components of the time-series possess the same frequencies (e.g., similar time of variation between the two state involvement goals). However, this method cannot be directly applied to the analysis of the phase synchronization between two plurifrequential components of a time-series. A plurifrequential time-series is composed of a time-series with multiple frequencies occurring in the same time as it is commonly the case in living systems. For instance while a pigeon is walking, if we observe the movements of the head, we would notice two frequencies: one frequency triggered by the pigeon’s gait and a second one caused by the rhythmical forward and backward movements of the head, commonly called head-bobbing (Frost, 1978; Green et al., 1994; Troje and Frost, 2000). Let’s consider another example based on the dynamics of self-esteem (Ninot et al., 2005) to illustrate the idea of plurifrequential time-series. These authors studied the dynamics of self-esteem over a 6-months period. The results revealed a short history dynamic adjustment of self-esteem, which corresponds to frequency modulations of time-series in a short period of time (such as per day). They also discussed the possibility of another dynamic of self-esteem over a longer period of time. This hypothesis suggests the common presence of short-term and long-term dependencies of self-esteem. In other words, participants have demonstrated short-term adjustments of their self-esteem (time-scale = day) imbricated within long-term adjustments (time-scale = month-year). This indicates plurifrequential components in the self-esteem time-series. The CWT method would be able to deal with such plurifrequential time-series and is conjointly able to detect the phase synchronization of such time-series. The next section will be dedicated to an explanation of the interrelation between the notion of frequency and the notion of RP in time-series, which will facilitate an understanding of how the CWT method works.

### THE RELATIVE PHASE AND FREQUENCY RELATIONSHIP

A simple way to illustrate the interdependence between the notion of frequency and RP is to use a practical example – an enacted series of knock-knock jokes. In this dual situation, most of the sentences are controlled and set. For example, the joke always starts with the teller saying: ‘knock-knock’ (first utterance) and the responder answering ‘who’s there?’ (second utterance). The main objective for Schmidt et al. (2012) was to understand better the behavioral synchronization occurring in social interaction during structured conversation. Their findings revealed the presence of two “behavioral waves” (i.e., two frequencies) when analyzing a series of 10 consecutive jokes: one of these frequencies corresponding to the time-scale of the utterances and the other one corresponding to the time-scale of the joke. A synchronous synergy (i.e., RP) was observed between the two participants when looking at the utterances time-scale (in-phase). When looking at the joke time-scale, a lag was observed between the teller and the responder with the teller leading the behavioral interaction (RP ≈40°). This experimentation illustrates that, in the context of social interaction, the behavior can be nested in several temporal scales (plurifrequential time-series). Each of these scales contributes to the emergence of human communication interaction. In this example, the interaction required 0° of phase on one frequency and 40° of phase on the other frequency. The joint analysis of both time (frequency) and space (RP) provides the foundation to better understanding the structure of coordinated actions.

As illustrated in the above example, the notions of RP is intrinsically related to the notion of frequency. Understanding the time-series characteristics is essential in the identification of the frequency (or the period) components and consequently crucial when it comes to analyzing the RP (Fuchs et al., 1996; Sternad et al., 1999a,b; Rosenblum et al., 2001; Peters et al., 2003; Pikovsky et al., 2003; Kano and Kinoshita, 2010). In that sense, the CWT method takes into account both frequency and RP components of the analyzed time-series.

## WHY DOES IT SEEM IMPORTANT TO USE THE CROSS-WAVELET TRANSFORM METHOD?

As discussed above, classical methods (DRP and Hilbert transform) break down when the time-series are composed of two or more main frequency components [plurifrequential time-series – see examples on (i) short-term and long-term adjustment of the self-esteem or (ii) the knock-knock jokes]. Such situations are well suited to the CWT method because it does not require any hypothesis about the nature of the time-series. Complex time-series having frequency, amplitude, and/or phase modulations do not prevent the use of the WT method. The analyzable time-series could evolve in a random way without any consequences on the results. It would typically be the case if we had to analyze the dynamic of self-esteem. The short-term dynamic adjustment can appear and disappear over time without influencing the analysis of the long-term dynamic adjustment (Ninot et al., 2005). The two levels of analysis can be performed independently. Therefore, the frequency, amplitude or RP modulations at one level do not affect the results of the other levels of analysis. Moreover, having the possibility to get the RP for each of the frequencies provides complementary information. The RP results for the knock-knock experiment at the joke time-scale offers a better understanding of the leader-follower relationship with a specific quantification of the time-lag between the joke teller and the responder. Each of the frequency and RP characteristics specific to the CWT method will be discussed in the section “HOW.” The objective of the following section is to highlight WHO is currently using the CWT method and to whom it could be extended to in several psychological domains.

## WHO IS USING THE RELATIVE PHASE AND THE CROSS-WAVELET TRANSFORM METHOD?

The CWT method has been applied in diverse scientific domains as mathematics, engineering science and more recently in physiology (Sakowitz et al., 2001; De Carli et al., 2004; Rezaei et al., 2008) and neuroscience (Herreras et al., 1994; Rodriguez et al., 1999; Armand and Minor, 2001; Cao et al., 2010; Westin et al., 2010). In psychology some studies used the WT method to examine verbal or non-verbal sounds (Rendalla et al., 2007; Uclés et al., 2009), recognition of emotional facial expression (Knyazev et al., 2008) or interpersonal motor coordination (Issartel et al., 2007; Ashenfelter et al., 2009; Schmidt et al., 2014). The interested readers will find resources introducing the CWT method in methodological articles or books (Chui, 1992a,b; Daubechies, 1992; Burrus et al., 1998; Torrence and Compo, 1998; Mallat, 1999; Walker, 1999; Percival and Walden, 2000; Le Van Quyen et al., 2001; Addison, 2002; Fugal, 2009).

### WHO COULD USE THE RELATIVE PHASE AND THE CROSS-WAVELET TRANSFORM METHOD?

The RP offers several opportunities to investigate time-series in psychology. For this a reason, RP could now be used in many psychological fields to characterize human behavior. It could be applied to different kinds of human phenomena from synchronization between systems within a single human (e.g., locomotor respiratory coupling, Villard et al., 2005), to inter-limb co-ordination (e.g., the index finger of the two hands, Haken et al., 1985), or interaction between personality characteristics such as self-confidence, performance goal flow (Gernigon et al., 2002 – see detailed explanation in Section “Relative Phase”). The latter research group have analyzed the dynamics of psychological momentum from an actor or an observer perspectives in order to deeply investigate the moment-to-moment variations of a given variable (Briki et al., 2013, 2014). Using the CWT method, this research group could have reached similar goals while also being able to perform a complementary series of experimentation. The positive and negative psychological momentums could be manipulated and consequently compared to the dynamics of affective or motivational components. Along the same line, the study by Avugos et al. (2013) investigating the role of self-efficacy on athletic performance could benefit from the current method as they could analyze the interaction between performance outcomes and self-efficacy beliefs over time. Other application domains could also be found in sport or in daily situations at work between a line manager and employees for example. It seems also important to consider the dynamics of learning as a potential application area for the CWT method. For example, the relationship between developmental dynamics of mathematical performance (Aunola et al., 2004) and cognitive, motivational, or achievement-related task-avoidant behavior (Hirvonen et al., 2012) variables. In the context of social psychology, the relationship between a buyer and a supplier for example can be considered from the cyclical attraction process angle (Ellegaard, 2012). Industrial marketing research is interested in uncovering the features and process of attraction to understand better the close ties between customers and suppliers. In this context, the CWT method could contribute in quantifying specifically this interpersonal relationship in analyzing people behaviors or communication strategies over time and observed whether there were several levels (that we call frequencies here) that triggered different behaviors. Social psychologists have also investigated dyadic situation in the development of antisocial behavior (Granic and Lamey, 2002; Granic and Patterson, 2006). Using the space space grid (SSG), they were able to quantify, in real time, observational data of two participants (Lewis et al., 1999). This methodology allowed them to identify specific conditions drawing the dyadic behavior toward hostile or cooperative behavior. An analysis of these oscillatory behaviors over time with the CWT method would be complementary to this analysis (e.g., hostile ≈180° and cooperative ≈0° of RP) with a quantification of the time lag (or latency) between the two participants behavioral response as well as an estimation of the speed of the response.

More widely, the CWT could also be applied in psychiatry to observe mental disorders as altered neuronal organizations in the brain (Peled, 2005), or to give insight into chronic schizophrenia (Slewa-Younan et al., 2004) or even in interpersonal motor coordination to understand how two people synchronize with each other (Schmidt et al., 1998; Richardson et al., 2005). In light of the above, other fields in psychology not currently using the RP and/or the CWT method may see the purpose of doing so in the future (Riley and Holden, 2012). The next section will be dedicated to the question of “WHEN” in order to exemplify the kind of data that should be considered as suitable for the CWT method.

## WHEN CAN WE USE THIS METHOD?

### CATEGORICAL vs. CONTINUOUS SCALES

Categorical data can be sorted according to a discrete category. The first type of categorical data is nominal data. Nominal data is named or labeled without any regard to the value of the observation. In a nominal set of data the values associated with the observation are not measured. For this reason, nominal data cannot be used with the CWT method. The second type of categorical data is ordinal data. An ordinal set of data has a natural order, therefore the values can be ranked but the difference between neighboring points on the scale may differ. The CWT method can only be applied on a set of data having constant differences/intervals between two adjacent observations.

Continuous data scales refer to scales where the interval between observations (sampling rate) is constant. All kinds of continuous data can be analyzed with the CWT method.

### WHAT HAPPENS IF DATA ARE MISSING?

The WT and the CWT cannot be applied in the case of missing data in a time-series. Either data have to be re-collected or imputation methods can be used to fill in the missing data. At this stage, one need to identify what are the best imputation methods to use. With regard to this, an extensive literature is available (for a review see Little and Rubin, 2002). The authors strongly recommend extreme caution in the use of any kind of imputation strategy keeping in mind the possibility of potentially biased interpretations.

### ARE THERE DURATION BOUNDARIES FOR THE ANALYSES?

The minimum duration of the time-series depends on the lowest frequency (longest period) of the time-series (i.e., the slowest set of data to be analyzed). Based on this knowledge, the minimum duration of the time-series analyzed is twice the duration of the lowest frequency. Let’s take the example of a study on the dynamic of self-esteem with the assumption that the long-term modulations correspond to a period of 2 months. Therefore, one should measure the dynamic of the self-esteem once a month during 4 months (see Minimum Sampling Rate). In terms of the maximum duration of the time-series to analyze, there is mathematically no boundary.

### MINIMUM SAMPLING RATE

The minimum sampling rate (or sampling frequency) recommendation is based on the Nyquist–Shannon sampling theorem. The lowest bound for the sampling rate has to be two times the highest frequency of the time-series. For example, if the fastest behavior observed occurs every 2 s then the highest frequency is 0.5 Hz (Frequency = 1/Period). Therefore, two times the minimum sampling rate of 0.5 Hz is 1 Hz. To illustrate this point, we could take the example of the enacted knock-knock experimentation from Schmidt et al. (2014). The highest observed frequency for this experiment was based on the utterance time-scale (i.e., 1.33 Hz). Based on the Nyquist–Shannon sampling theorem, the minimum sampling rate should be 2.66 Hz. The actual sampling rate they used was almost six times faster (i.e., 15 Hz). In this situation, this sampling rate guarantees a fine temporal accuracy for the RP analyses. Overall, the sampling rate should be maintained throughout the duration of any given experiment. If it is not the case, interpolation methods should be used.

### DOES THE SAME SET OF CRITERIA APPLY FOR CWT AND WT METHOD?

For both the WT and CWT methods, the same criteria apply. For a valid comparison between two time-series, the CWT analysis requires the same duration for the two time-series and a similar sampling rate. Downsampling methods may be useful if one of the two time-series has a higher sampling rate than the other.

The next section will be dedicated to a step-by-step description of HOW it is possible to use the CWT method.

## HOW TO USE THE WAVELET TRANSFORM METHOD

This step-by-step presentation of the WT will explain the key elements of the WT method in a comprehensive treatment. The mathematical aspect of the method is not in the scope of this tutorial. The description will allow the reader to understand the elements in order to apply the method him/herself. When the reader has an understanding of the following keys elements, he/she will be ready to apply this method on their own signals using the wavelet toolbox of the Matlab software (MathWorks Inc., Natick, MA, USA). Readers can also use the code currently utilized for the analyses in this article as well as a “Cook-book” on how to use and understand the code. The code can be downloaded at the following address: ‘http://webpages.dcu.ie/~issartej/WavePackage.zip’

### INTRODUCTION TO SPECTRAL METHODS

The Fourier transform (FT) is a method transforming a time-series from the time domain to the frequency domain. This spectral analysis provides a global description of the frequency content of a given time-series (for more details on the application of the FT method in psychology see Vallacher and Nowak, 1994). When we want to understand when (temporal dimension) an event occurs in a time-series, the analysis in the frequency domain independently of the temporal domain cannot provide a satisfactory answer (Flandrin, 1988). Consequently, methods have been developed to take into account both time and frequency content of a time-series. The most logical and well-known method is the Windowed FT (WFT) based on the FT method itself. But instead of analyzing the full time-series, the FT is performed on short consecutive (overlapping or not) segments. The moving/translating windows allow for the detection of sudden changes in the frequency content of the time-series. The main limitation of this method is the lack of precision to either the time or the frequency domain. The size of the segment will determine either a high level of precision in the time domain or in the frequency domain. For example, a small window would not allow for the detection of any event larger than the window while maintaining a good localization in time. On the other end, a large window will take into account the long-term event (frequency domain) but with a high level of imprecision in the temporal domain.

A further methodological development of the WFT led a way to compare two time-series together. The cross-WFT (CWFT) can evaluate the interactions between 2 time-series providing information about the common frequency content between them as well as the difference in phase at a given frequency. It is important to note that the limitation presented above for the FT method also apply to the CFT method. Overall, highlighting these two methods is central to this article, as they constitute the foundation stones of the further development of WT and the CWT^3^.

### ORIGINS OF THE WAVELET TRANSFORM METHOD

Historically, the WT method was introduced in seismic research by Morlet (1983). Since then, wavelets are commonly used in geosciences (see Kumar and Foufoula-Georgiou, 1997 for a review) as they are particularly well-suited in characterizing the “local” properties of time-series. The meaning of “local” has to be understood as the opposite of “global.” As mentioned in the previous section, the “global” properties of a time-series refer to an average/summary of the time-series properties not taking into account the evolution of these properties over-time. The WT method will characterize the “local” properties of the time-series. Each occurrence of the time-series will be taken into account in the analysis. Due to these properties, the WT has been the object of various methodological developments and numerous applications in scientific domains as detailed in the section WHO.

### FUNDAMENTAL KEY STEPS OF THE WAVELET TRANSFORM METHOD

The WT method is based on a FT analysis. As discussed in Section “Introduction to Spectral Methods,” the FT is not able to detect temporal discontinuities or yield information on the temporal persistence of periodicities. The conjoint characterization and quantification of each frequency component in time must be taken into consideration as does the characterization and the quantification of the associated RP. This joint characterization of the frequency content of the time-series in time while keeping a high level of precision in both time and frequency domains constitutes one of the WT advantages. The exploration of the frequency range of interest as a function of time results in a “time-scale” or time-frequency representation (2D map) of the time-series. This time-frequency representation could be compared to a piece of music (that represents the time-series) written as a musical score. Each musical note could be represented by a frequency parameter (y-axis) and by the time of occurrence (x-axis). Before, we go into a deeper explanation of the method, we will explain what a time-frequency representation (or time-frequency plane) is.

### TIME-FREQUENCY PLANE

**FIGURE 1:**
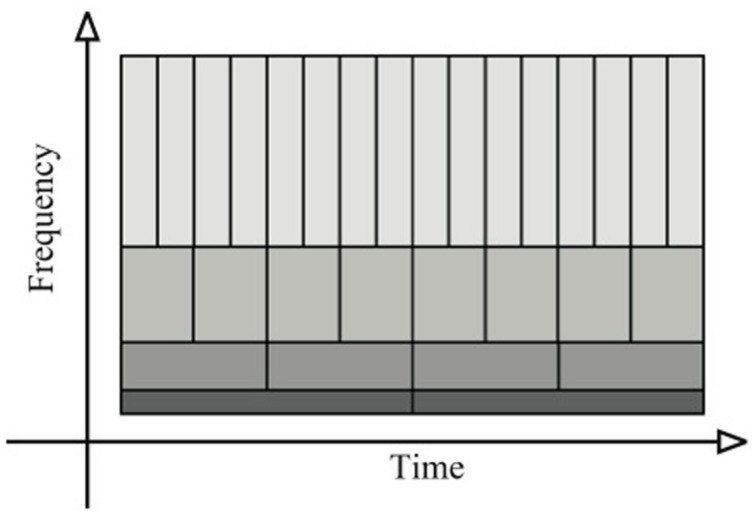
**Tiling of the time-frequency plane for the wavelet transform (WT) method.** Narrow rectangles are used for the high frequencies that give a precise localization in time. Large rectangles are used for the low frequencies that give a precise localization in frequency. This illustrates the trade-off between the accuracy in time and the accuracy in frequency.

The time-frequency plane is the graphical representation used to illustrate the results obtained with the WT and the CWT method. This time-frequency plane contains the same information as the original time-series but expressed in another way: in frequency as a function of time. The time-frequency plane is defined by (i) the frequency that ranges from zero to the Nyquist frequency (i.e., 1/2δ(t), where δ(t) is the sampling interval – see section WHEN) and (ii) the time spans of time-series. As illustrated in **Figure 1**, the time-frequency-plane is tiled with rectangular tiles, usually called Heisenberg cells. The area of each cell is dictated by the uncertainty principle. This principle illustrates the necessary trade-off, in the calculations, between the accuracy in time and in frequency that influence consequently the accuracy of the phase calculation. A better resolution in the time domain triggers a diminution in the frequency resolution and vice-versa.

**FIGURE 2:**
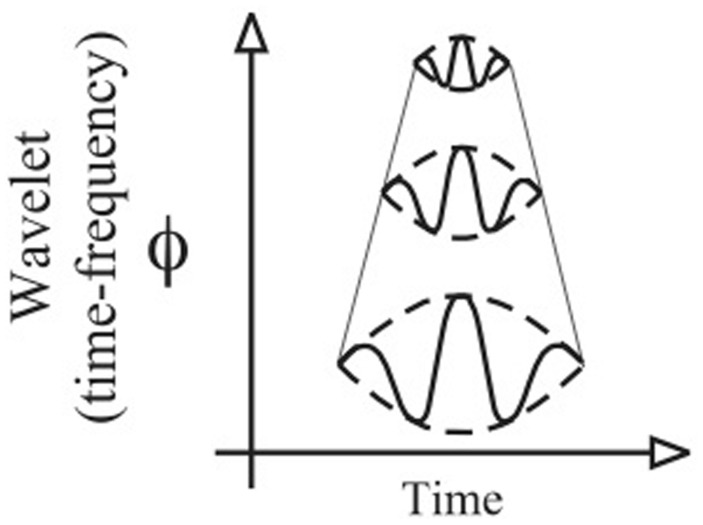
**Representation of the contraction/dilatation of the analyzing function in the time-frequency plane**.

For the study of the WT, Flandrin (1988) called the time-frequency plane a scalogram. In a scalogram, the time-scale of the rectangle is flexible (c.f. **Figure 1**). Narrow rectangles are used for the high frequencies that give a precise localization in time. Large rectangles are used for the low frequencies that give a precise localization in frequency. Thus, the scalogram possesses multi-scale structures that allow us to precisely define the temporal and the frequency locations and to perform a multi-scale analysis (Torrence and Compo, 1998). Each of these structures contains an analyzing function (details about this function will be provided below) used during the computation to analyze the properties of the time-series. The modification of the size (dilation/contraction) of this analyzing function over time (translation) provides the key in quantifying the frequency and the RP location of the time-series (**Figure 2**).

### DILATATION/CONTRACTION/TRANSLATION OF THE ANALYZING FUNCTION

The WT is calculated by convolving the time-series s(t) with an analyzing wavelet function ψ(a,b) (derived from a mother function ψ) by dilatation of a and translation of b. The variable *a* is the scale factor that determines the characteristic frequency so that varying *a* gives rise to a spectrum; *b* is the translation in time so that variable *b* represents the “sliding window” of the wavelet over s(t) – see **Flowchart** (step 2). Concerning the scale factor *a*, the analyzing wavelet function is swept over the whole time-series for the frequency ranges as a function of time. Because the WT method is naturally bound and invariant by translation, it is particularly suited for a local analysis. According to the frequencies analyzed, the analyzing function is either dilated or contracted. Thus, the tiling of the time-frequency plane depends on the contraction/dilatation of the analyzing function (**Figure 2**). Hence, the WT method is also invariant by contraction/dilatation, allowing multi-scale analysis (**Figure 2**). These properties offer a good trade-off in both time and frequency domains (Torrence and Compo, 1998; Percival and Walden, 2000).

**FLOWCHART:**
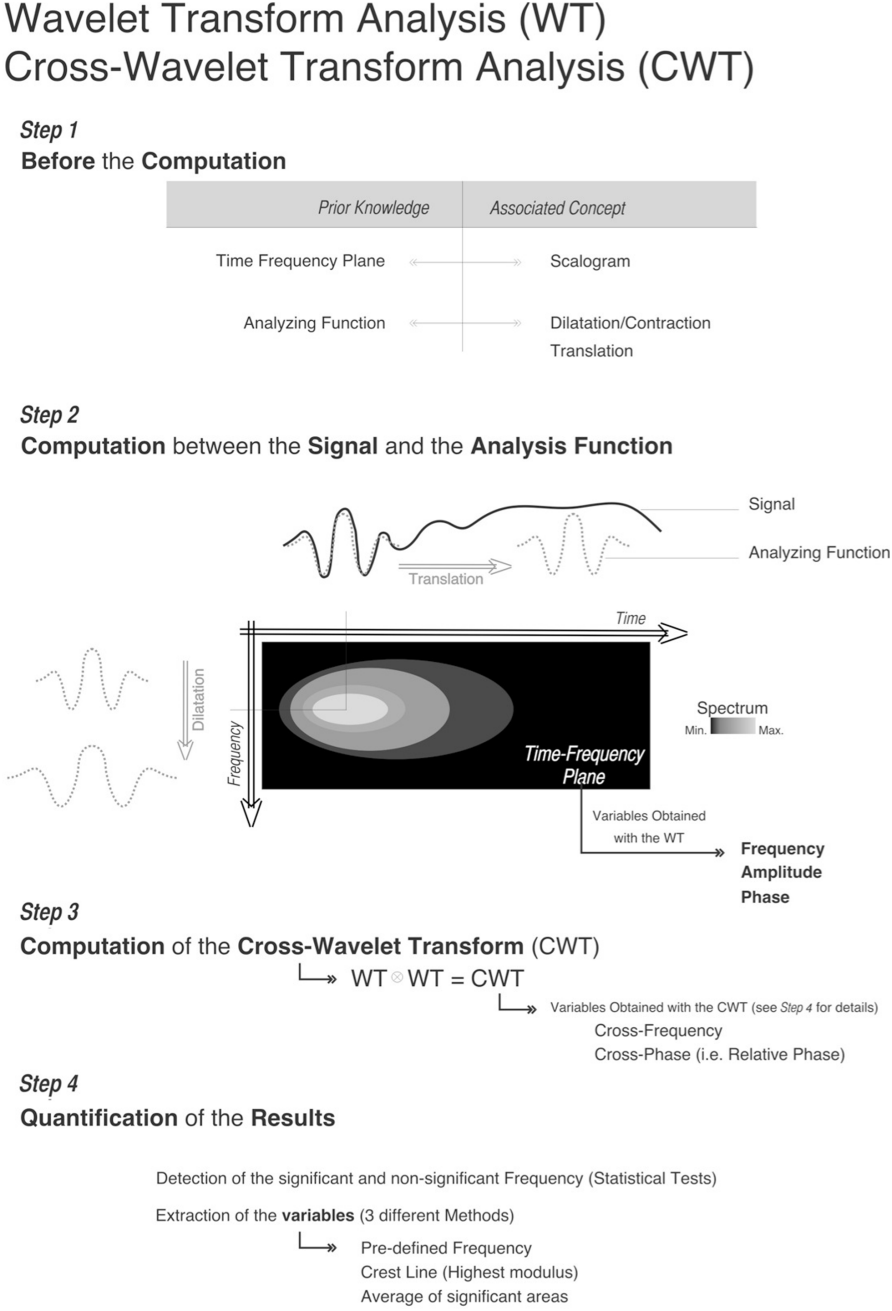
**Flowchart of the WT and CWT method computation**.

**FIGURE 3:**
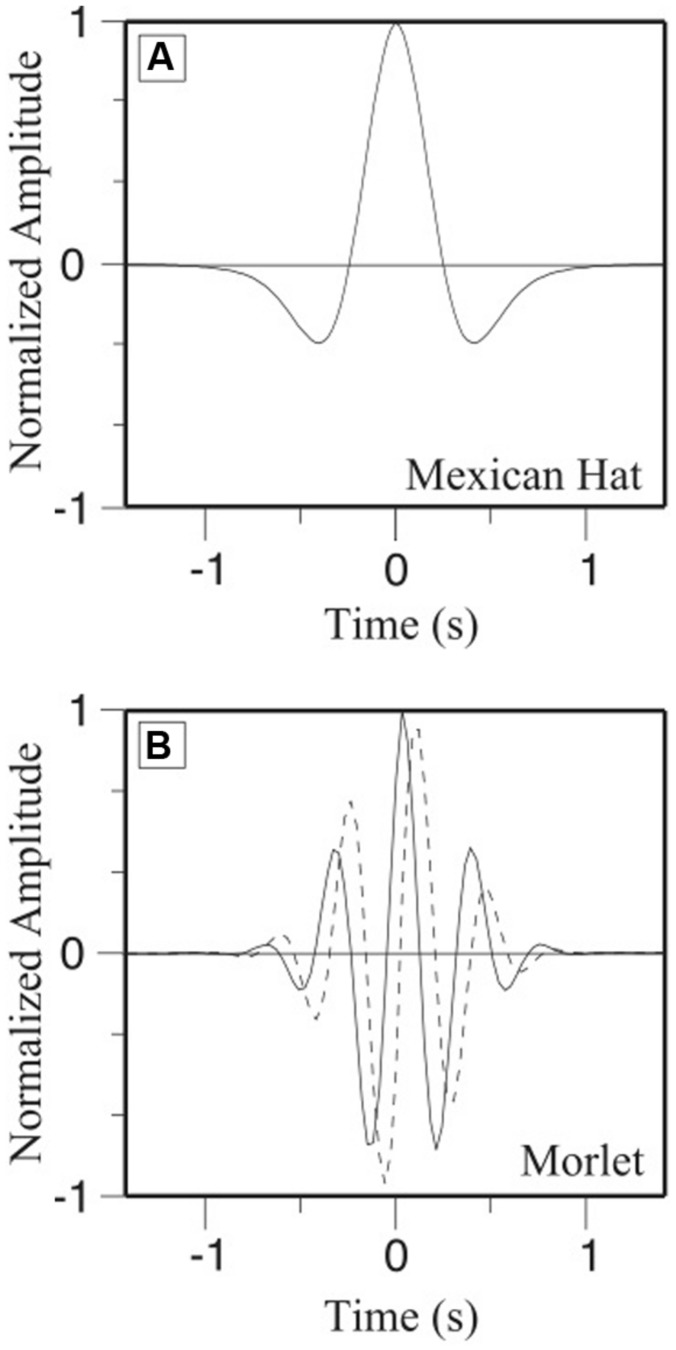
**Temporal representation of **(A)** the Gaussian (Mexican Hat) and **(B)** the Morlet analyzing functions**.

Mathematically a great number of analyzing functions (see examples **Figure 3**) could be created (Torrence and Compo, 1998). The choice of the analyzing function is neither unique nor arbitrary and mostly dependent on the likeness between the time-series and the analyzing function. Specific descriptions and recommendations of the properties of the analyzing function can be found in the literature (e.g., Torrence and Compo, 1998; Mallat, 1999; Fugal, 2009). We recommend to the interested readers to refer to this literature before starting the analysis of their time-series.

**FIGURE 4:**
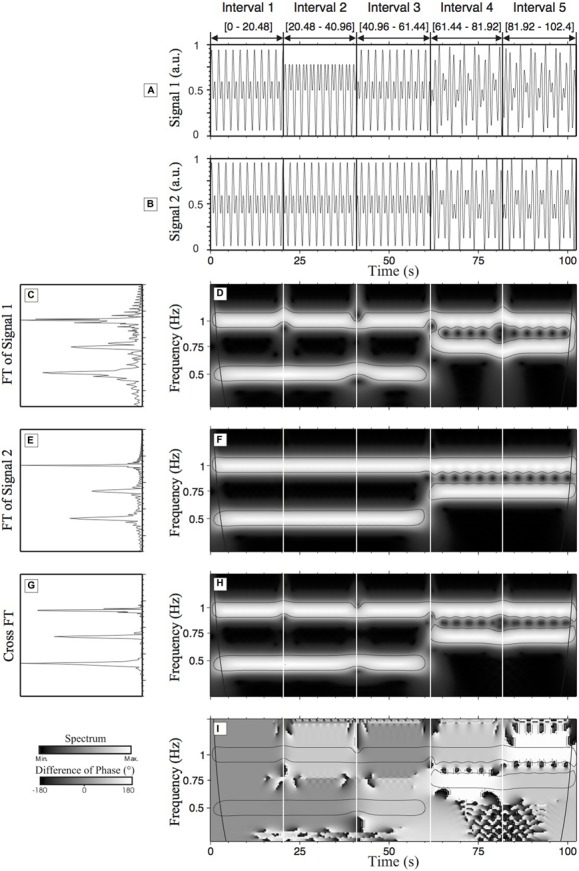
** Wavelet transform analysis and cross-wavelet transform (CWT) analysis of time-series (s_**1**_ and s_2_). (A,B)** are the representations of s_1_ and s_2_, respectively; **(C,E)** show the normalized Fourier spectrum of **(A,B)**, respectively. In both cases, we can observe one main peak at 1 Hz and two other peaks at 0.75 Hz and 0.5 Hz. **(D,F)** are WT spectra (scalograms) of **(A,B)** respectively. These figures help to localize and quantify in terms of time of localization (time) and amplitude the frequency components previously identified in the Fourier spectra. In **(D,F)** two frequency components are present in the three first intervals of the time-series s1 and s2: one frequency at 1 Hz and one at 0.5 Hz. In the two last intervals, there are two frequencies: the frequency at 1 Hz, as in the three first intervals, and a new intermediate frequency at 0.75 Hz. **(G)** shows the cross-Fourier spectrum of **(C,E)**. **(H)** Shows the cross-wavelet spectrum that is a representation of the common frequencies of **(D,F)** reflecting the local degree of interaction between the two analyzed time-series s_1_ and s_2_. **(I)** Shows the difference of phase of **(A,B)**, i.e., the difference of phase between s1 and s2. The levels of gray permit us to visualize the associated relative phase (RP). a.u., arbitrary unit.

The best way to understand these principles is by examining an example based on easily understandable time-series. To do so, two synthetic time-series were created. The two time-series, s_1_ (**Figure 4A**) and s_2_ (**Figure 4B**) have a duration of 102.4 s (with a sampling rate of 50 Hz). The amplitude of s_1_ and s_2_ is 1 arbitrary unit (a.u.). Both time-series have been divided into five intervals. For the first three intervals [0–61.44], the time-series are composed of a high frequency component at 1 Hz and a low frequency component at 0.5 Hz. For the last two intervals [61.44–102.4], the time-series are composed of a high frequency component (1 Hz) and an intermediate frequency component (0.75 Hz). The first interval is characterized by a zero degree RP between s_1_ and s_2_ (the two time-series are identical in this interval). In interval 2, the time-series s_1_ is characterized by a 90° phase lag in the high frequency component (1 Hz) whereas a 90° phase lag is applied on s_1_ in the low frequency component (0.5 Hz) of the third interval. In the fourth interval, a phase lag of 90° on the high frequency component and a phase lag of 180° on the intermediate frequency component are applied to s_1_. In the fifth interval, a phase lag of 180° on the high frequency component and of 90° on the intermediate frequency component are applied to s_1_ (see **Table 1** for a summary of the changes made to s_1_). Note that no phase lag exists in s_2_.

**Table 1:** Details of the phase lag and frequency modulations of s_**1**_.

	**Interval 1 [0-20.48]**	**Interval 2 [20.48-40.96]**	**Interval 3 [40.96-61.44]**	**Interval 4 [61.44-81.92]**	**Interval 5 [81.92-102.4]**
**s_1_**					
High frequency = 1 Hz	Φ = 0°	Φ = 90°	Φ = 0°	Φ = 90°	Φ = 180°
Intermediate frequency = 0.75 Hz	∅	∅	∅	Φ = 180°	Φ = 90°
Low frequency = 0.5 Hz	Φ = 0°	Φ = 0°	Φ = 90°	∅	∅

*Φ represents the value of the phase lag and ∅ represents the interval where the frequency associated is not present in the time-series.*

The above description of the synthetic time-series could be potential hard to relate to a “real” example. To facilitate the reading, we propose to describe this time-series as if it was a moment-to-moment and/or a day-to-day dyad interaction between a parent and a child. This dynamical interaction constantly evolves with several time-scales: from short-term interactions (e.g., dual tasks) to long-term interactions (i.e., week-to-week, year-to-year). On this basis, interval 1 would represent a high synergy (0° RP) between the parent (s_1_) and the child (s_2_). The shift to a 90° degree RP in interval 2 would be associated with some sort of latency from one of the participants in reaction to the other participant’s behavior. In an ongoing situation, when an unpredicted event occurs, it induces a dysregulation of the interaction. This could trigger anger or sadness to a child. In this context, the latency (90° RP) could be explained, for example, by a delayed response/reaction of the parent to the child’s anger. After a given time (interval 3), the short-term interaction between the parent and the child goes back to a high level of synchrony (0° of RP) with a knock-on effect on the long-term interaction (90° RP for the low frequency). Intervals 4 and 5 at the short-term interaction level indicates a deterioration of the relationship up to a point (180° of RP) where the parent and the child behaviors are going in the opposite direction. This behavioral shift could also be observed on the longer-time scale (Intervals 4 and 5). This seems to indicate the bidirectional nature of the interaction across the different time-scales. If we were to interpret these results, one will suggest that the short-term and long-term interactions are influencing each other with potential consequences for (i) the person itself (e.g., developmental outcome) and his/her environment (e.g., friends and family). In the following section, we will regularly refer to this example to illustrate the key aspects of the CWT method.

**Figures 4A,B** represent the amplitude of time-series 1 (s_1_) and time-series 2 (s_2_) respectively on the Y-axis as a function of time. **Figures 4C,E,G** represent the FT spectra of s_1_ and s_2_ respectively as well as the cross-FT. From these two types of representation (time vs. frequency), it is very difficult to quantify differences, similarities or interactions between these two time-series. By visually inspecting the time-representation of s_1_ and s_2_ (**Figures 4A,B**) it is clear that something happened at the beginning of intervals 2 and 4 (e.g., sudden modification of the nature of the interaction between the parent and the child), but it is impossible to relate these changes to modifications in the frequency dynamics. On the other hand, a comparison of the Fourier spectra (**Figures 4C,E,G**) informs us that s_1_ and s_2_ contain three main frequency components. But these spectra do not provide any information about how these frequency components evolve with time (i.e., when this sudden interaction changes occur). The WT time-frequency representation of the two time-series (**Figures 4D,F**) provides both the temporal and frequency information. We can see such a representation in **Figures 4D,F** that represent the WT spectrum of s_1_ and s_2_ respectively.

The previous explanations constitute a necessary first step in the understanding of (i) the WT method and more specifically (ii) the time-frequency representation. The information contained in the time-frequency representation has to be quantified in order to perform statistical analysis.

### STATISTICAL TEST TO DISCRIMINATE THE PROPERTIES OF TIME-SERIES (SEE **FLOWCHART** STEP 4)

Hence, from the CWT method, we obtain an expression of the whole range of frequency as a function of time. In order to quantify and use the results of the WT, statistical analysis can be applied. The first objective is to determine the frequencies statistically present in the WT spectrum (also called a scalogram). The statistical test used in this article is based on the test used by Torrence and Compo (1998). These authors have demonstrated that, each point of the WT spectrum is statistically distributed as a chi-square with two degrees of freedom. The confidence level is computed as the product of the background spectrum (the power at each scale) by the desired significance level from the chi-square (χ^2^) distribution. When the WT spectrum is higher than the associated confidence level it is said to be “statistically significant.” If the background spectrum is not known, one should use, as recommended by the latter authors, the global wavelet spectrum (time-average of the wavelet spectrum) as background. By extension, Torrence and Compo (1998) established the confidence levels of a cross-wavelet spectrum (see next section) from the square root of the product of two chi-square distributions. The confidence level is determined classically at the desired level (in this article we used 95% confidence). The set of statistically significant coefficients is a matrix of numbers that it is possible to extract for further quantitative analysis.

In **Figures 4D,F,H,I** the thin black lines outline statistically significant zones defined from the statistical test proposed by Torrence and Compo (1998). As we previously explained, the WT permits us to detect the time where the frequency modification appears. From the top to the bottom of **Figure 4D**, it is clear that in the three first intervals [0–61.44] we can see, firstly a white band that represents the high frequency component at 1 Hz (short-term adjustments) and secondly the white band that represents the low frequency component at 0.5 Hz (long-term adjustments). For the first three intervals, **Figures 4D,F** are exactly the same except at the beginning of each interval for **Figure 4D** due to influences of the phase lag (see **Table 1**) on the frequency values. To refer back to the example here, this suggests that the parent’s behavioral latency has an effect on both short-term and long-term adjustments. The modifications of the phase lag at the beginning of the intervals of time-series 1 reorganize temporarily the frequency values of each frequency component (the white band is not centered around 1 Hz or 0.5 Hz). These modifications illustrate the accuracy of the WT method in detecting frequency modifications occurring in a time-series. Moreover, in **Figures 4D,F**, we can accurately detect, between intervals 3 and 4, a modification of the frequency component of the time-series represented by the end of frequency component 0.5 Hz and the beginning of frequency component 0.75 Hz (i.e., change in the parent time-scale from long-term adjustments to middle-term adjustments).

So far, the discussion of **Figure 4** has been orientated on the graphical representation obtained from one time-series (the WT method). The next section will be focused on an explanation of the methodological characteristics of the cross WT method.

### CROSS-WAVELET TRANSFORM AND RELATIVE PHASE (SEE **FLOWCHART** STEP 3)

The WT method can be extended to the analysis of the interactions between time-series and to the calculation of the RP, as we previously mentioned. The CWT is computed from the WT of each time-series (e.g., *f* and *g*) *W_f_* and *W_g_*. The CWT method of the two time-series *W_fg_* is the product of *W_f_* with the complex conjugate of W_g_. The time-frequency representation of the cross-wavelet modulus gives information about the intensity of the interaction between two time-series for each frequency (i.e., time-scale) as a function of time (the cross-wavelet modulus). Moreover, the CWT also allows us to access the continuous RP of two time-series for each of the main frequencies (Daubechies, 1992; Liu and Chao, 1998). Two or more common independent main frequency bands may be detected with an associated RP for each frequency band (i.e., type of interaction and degree of synergy for each of the time-scales). The temporal evolution of the frequency may be detected as well as the associated RP (magnitude of the behavioral latency between a parent and a child reactions).

Going back to the synthetic example, **Figures 4H,I** represent the cross-spectrum and the cross-phase (the RP) of s_1_ and s_2_ respectively. Focusing on interactions between s_1_ and s_2_, as discussed above, the cross Fourier spectrum (**Figure 4G**) does not provide enough information about the synchronization processes between the two time-series as all temporal information is lost (i.e., when does a behavioral shift occurs in the dyad?). The combined analyses of the cross-spectrum and cross-phase reveal, in a quantitative form, the frequency interactions (common time-scales between the parent and the child) and RP between these two time-series as a function of time and frequency. Common frequency components that span common time intervals can be identified in the white bands (**Figure 4H**) and the RP for those components can be directly read from **Figure 4I**. With the CWT, the RP between s_1_ and s_2_ is not averaged over time or frequency, and so, provides major advances in respect to the former approaches. In the first interval of **Figure 4I**, the spectrum in dark gray (inside the significant area) represents a RP of 0° between the two time-series for the two frequency components (i.e., high behavioral synergy). In the second interval, we can see the RP of 90° (light gray) on the high frequency component (1 Hz – short-term adjustment) and the RP of 0° on the low frequency component (0.5 Hz – long-term adjustment). We can observe in interval 3, the RP lag of 90° on the low frequency component (0.5 Hz) that is represented by a light gray color. Altogether, the results of intervals 2 and 3, clearly illustrate the ability of the CWT to locally detect the RP for each component of the time-series (i.e., the behavioral latency for each of the time-scale). In interval 4, the modification of the frequency component (disappearance of the frequency of 0.5 Hz and emergence of the frequency of 0.75 Hz) does not influence the computation of the RP using the CWT approach. We can precisely detect the RP of 90° (light gray color) on the high frequency (1 Hz) component and a RP of 180° (white color) on the intermediate frequency component (0.75 Hz). This modification of the time-scale associated with an important shift of the RP is characteristic of a dynamical change in the dyad (i.e., child and parent behavior are now going in the opposite direction). Similar observation can be done in interval 5. On the high frequency component (1 Hz), there is a RP of 180° (white color) and on the intermediate frequency component (0.75 Hz) a RP of 90° (light gray color). This example illustrates that the CWT method can detect the phase synchronization that could occur in plurifrequential time-series (i.e., several time-scales occurring at the same time). These illustrations are mostly based on a visual description of **Figure 4**. The next step is to understand how to extract data from the time-frequency representation (for instance from **Figures 4D,F,H,I**).

### HOW TO EXTRACT USEFUL INFORMATION FROM A TIME-FREQUENCY REPRESENTATION (SEE **FLOWCHART** STEP 4)

The following explanation will facilitate the choices to make before extracting the data. These descriptions will be based on a simulation of extraction of the data obtained from the CWT methods on time-series s_1_ and s_2_ (**Figures 4H,I**). The information that we need to extract is present in the cross-modulus (**Figure 4H**) and the corresponding difference in the phase scalogram (**Figure 4I**). The significant values are defined inside the zones outlined by the thin black lines. In this current section, we address the methods required to extract the necessary and interesting information from the significant areas. We will propose three methods. Each of them possesses specific properties. As a function of the data obtained and as a function of the experimental protocol performed, the experimenter will be able to make a choice between the different methods.

*First*, one could take the values of the cross-modulus and of the RPs that correspond to the frequency of movement imposed by the experimenters (in the case of the time-series s_1_ and s_2_, it corresponds to the frequency defined arbitrarily at the creation of these time-series). This choice requires a major hypothesis about the frequency values produced by the participants and on the manipulation of the frequencies by the experimenters. For each step, one could extract the cross-modulus and the RP at the frequency of movement imposed by the experimenters. Such a method can be applied when the experimenters are interested in the properties of the behavior at a specific frequency known before the experiment. Nevertheless, even if the experimenters impose a specific frequency, it is obvious that the behavior of the participants would oscillate around the imposed frequency. Then some significant data could be skipped. We suggest using more flexible methods to extract the information.

In the *second method,* one unique value is taken out of the CWT spectrum that corresponds to the highest (or maximum) cross-modulus value (in the significant area) for each time step, also called the crest line. This value corresponds to the time and frequency where the two time-series show a high degree of interaction. In such a case, the extraction of RP values is required to match up the corresponding index (time and frequency) of cross-modulus spectrogram on the cross-phase spectrogram. Hence, if we are obtaining a crest line, for example, at the 31st second at 1.04 Hz from **Figure 4H**, the corresponding RP has to be extracted in **Figure 4I** from the same time: 31st second and at 1.04 Hz.

The *third method* is the average of the significant area of the cross-modulus and its corresponding value of the cross-phase (RP) at each time. This third method gives a global view of the behavior by summarizing the information of the whole significant area at each time. With this method, the value used is the mean of the significant area for a given time. The third method could be applied to describe the global pattern, a global behavior for a given time without a precise localization of the frequency components. The standard deviation of the significant areas can be extracted as well.

The use of these different methods depends on the kind of analyses required by the experimental protocol and by the scientific question being asked. Our purpose was to give different potentially useful routes to using the WT and CWT methods. The final choice will be dependent on the experimenters themselves. Therefore, the statistical methods that could be applied are exactly the same ones as those classically used in the scientific domain (independent sample Mann–Whitney U, comparison of slope, MANOVA, Chi2, etc …).

### CONCLUSION

In summ ary, “multi-scale” investigation of the interactions between synthesized time-series presents interesting perspectives for studying human interactions. The CWT method allows us access to the evolution of the intensity of the interaction between the two time-series for each frequency as a function of time. Moreover, the properties of the CWT method permit us to access the continuous RP of two time-series for each of the main frequencies. Two or more common main frequency bands can be independently detected with their associated RP. Due to its “local” and “multi-scale” properties, this method is well-designed to deal with any kind of frequency modulations, amplitude modulations, abrupt time changes or frequency changes or any overlap in time and frequency.

The following section will detail a practical illustration using the results obtained from a real-life set of data. It will show how to determine the different parameters of the WT and the CWT methods. In a step-by-step procedure, we will explain how to extract, quantify and run statistical tests on data analyzed by WT and CWT methods. Each step will be fully detailed in order to help the reader to use the methods on his or her own.

## DATA FROM REAL-LIFE APPLICATION

### INTRODUCTION

The aim of this section is to illustrate the practical use of the WT and CWT presented above. As illustrated previously, the CWT method can be applied to different kinds of time-series (see section “When”). The current set of data has been obtained from the portal page of the European Network of Excellence called HUman-MAchine Interaction Network on Emotions (i.e., HUMAINE4). The purpose of the HUMAINE Network of Excellence concerned the development of systems that can “register, model, and/or influence human emotional and emotion related-states and processes” as can be read in the project description^4^. Part of the research program involved the development of the set of TRACE programs (Queen’s University Belfast, Belfast, UK). The TRACE programs let observers track the emotional content of a stimulus as they perceive it over time. The raters trace the way aspects of emotionality fluctuate as they appear in human emotional expression (e.g., Cowie et al., 2005; McKeown et al., 2012). The raters move a pointer in real time between markers representing the extreme state of a particular emotional dimension. The raters were asked to record their impressions of the emotions expressed in the stimuli. They used a scale from zero to one to report their impression of a person’s emotions. ‘Zero’ on the scale corresponds to zero emotion (i.e., totally emotionless) and ‘One’ corresponds to emotion at maximum intensity. They watched a video of a person and judged how much emotion he/she was experiencing from moment to moment by moving the cursor to a position in the scale that they thought best represented the degree of emotion being experienced.

### METHODS

Forty eight video clips are available from the HUMAINE database. These clips show emotion in action in different contexts (static, dynamic, indoor, monolog, and dialog). For the purpose of this practical example, we randomly selected one of these clips for the analysis. The clip shows a male participant being interviewed about his experience on a reality survival show. The selected video clip has a duration of 119 s. Ten participants were recruited to rate the video clip (five women and five men). The TRACE program was set up at a sampling rate of 10 Hz. The raters watched this video and performed the rating task to rate the emotional state of the participant.

**FIGURE 5:**
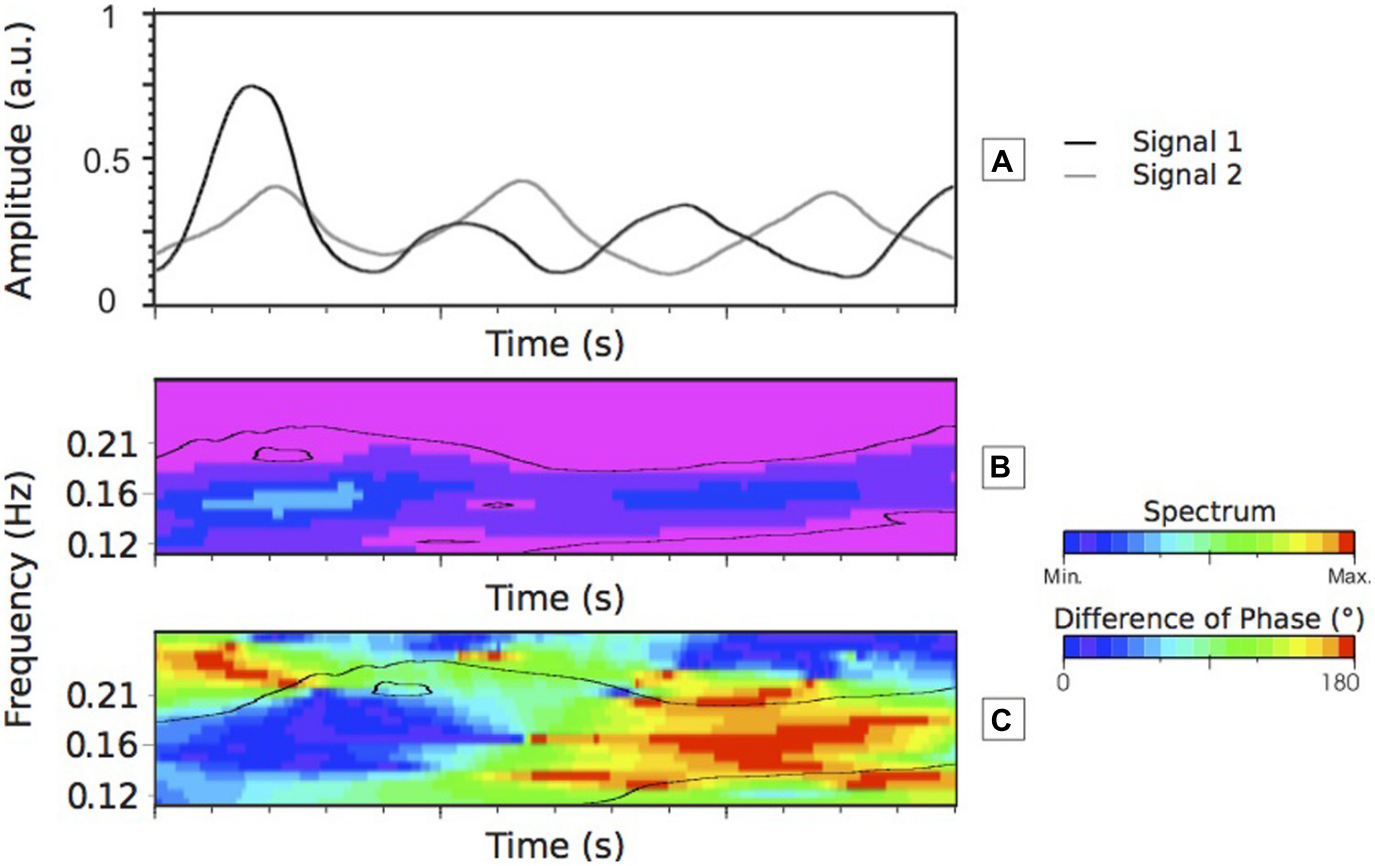
**Cross wavelet transform analysis of two males time-series (Signals 1 and 2). (A)** is the representation of Signals 1 and 2. The two signals seem to have a similar dynamic (similar frequency and similar direction) at the beginning but then while keeping a similar frequency the signals tend to be move in opposite direction. **(B)** Shows the CWT spectrum (scalogram) of Signals 1 and 2. The black lines delineate the area statistically significant of the common frequencies of Signals 1 and 2, reflecting the local degree of interaction between the two analyzed time-series. The highest spectrum value occurs at the beginning of **(B)**. This is mainly explained by the high amplitude (i.e., intensity) of Signal 1. Visually one can estimate the common frequency of the two signals around a value of 0.16 Hz. **(C)** Shows the RP between Signals 1 and 2. **(C)** Confirms the visual description of **(A)** where for the first part of the time-series, the two signals are closed to a phase pattern drifting to an anti-phase pattern toward the end of the time-series. The range of colors facilitates the visualization of the associated RP from in-phase (close to 0°) to anti-phase (close to 180°). a.u., arbitrary unit.

From this set of TRACE programs, one obtains a set of data where the amplitude of the trace illustrates the rise and fall of the rater “traces.” One can analyze the direction of changes, the intensity of changes or compare the dynamic of theses changes between participants, for example (see **Figure 5**).

Using the CWT methods, it is possible to evaluate the properties of the time-series and to estimate the individuals rating convergence and/or the divergence. Therefore the dynamic of the time-series can be compared. We can evaluate whether the raters consider that the emotional states of the participant are evolving at the same time (i.e., same speed of emotional state change). If they have the same dynamic, we will also be able to discern if the traces evolve in the same direction or not giving us some information concerning the nature of the rating (the RP) and its dynamic. **Figure 5C** represents the RP values between the two signals (**Figure 5A**). For example, one can be able to extract the continuous RP at a frequency of 0.16 Hz (see first method described earlier on in the HOW section). The RP values obtained for these given signals will be ranged from 0 to 180° RP over the duration of the time-series. The range of colors should help the reader in understanding the RP values. This will suggest that both raters judged at the same time an expression of the emotional state, but for one of the raters the emotional state rises and for the other one it falls suggesting that although the two raters have the dame dynamic (the same frequency), they go to an opposite direction (see **Figure 5C** where the values go from 0° – the two raters are in-phase – to 180° – the two raters are in anti-phase). In the next section, figures and statistical analyses will illustrate how we can extract some information on such time-series using the CWT method.

To identify the significant structures from a passive or noisy component of the CWT results, the Torrence and Compo (1998) method has been used [see HOW to Extract Useful Information from a Time-Frequency Representation (See **Flowchart** Step 4)]. As recommended by these authors, the desired level of 95% confidence has been applied.

### ANALYSIS AND DISCUSSION

The analysis of the time-series was performed with the Morlet analyzing function (Steps 1 and 2 of the **Flowchart**). This analyzing function is polyvalent in analyzing non-stationary time-series. To cover the frequency range of interest, a band of frequencies were chosen (0.05–1 Hz) for the whole analysis^5^.

Earlier on in the article we presented three different methods to extract the information provided by the CWT method. Regarding the analysis of the full set of data, we choose to apply the third method. The first method requires knowledge of the frequency to extract, which is not the case here since we are interested in the identification of the raters’ behavior. The second method was based on the highest values of the cross-modulus. This method could be used here but it may induce a bias. If two meaningful frequencies occur at the same time then this method will only be able to pick the highest, ignoring the second one. Therefore, the third method seems to be the most appropriate in this case. All characteristics of the time-series are taken into account from moment to moment. The value used is the mean of the significant area at each time.

The CWT method allows comparison between two-times series (step 3 of the **Flowchart**). The comparison between a group of people requires multiple comparisons that need to be explained to avoid any potential methodological confusion. For example, in the group of five females, each of them has to be compared with the four others. Then, for the 5 female participants A, B, C, D, E based on the CWT analyses we obtained 10 measures of the interaction between participants (i.e., AB, AC, AD, AE, BC, BD, BE, CD, CE, ⇔ DE 10 CWT). This leads to 10 comparisons to measure all interactions (common frequencies and RP) between participants.

For each of these 10 comparisons (step 4 of the **Flowchart**), we calculate the sum of the CWT spectrum for each frequency (CWT-F). From this, we obtain a distribution of the cross-frequency spectrum that represents the intensity of each frequency. The cross-correlation analysis of the CWT-F expresses the commonality between two participants in term of common frequencies and RP. This commonality is calculated with a cross-correlation analysis between each pair for each condition. Cross-correlations are calculated with Pearson product moment correlation coefficients. The statistical analysis performed on Fisher’s transform reveals the mean value of cross-correlation coefficients between pairs.

The cross-correlation analyses of the CWT-F provide a way to observe the interaction between the participants. The comparison two-by-two of the 10 CWT-F results in 45 comparisons. With all possible comparisons, we could potentially compare results from the CWT-F of participants AB with the CWT-F of participants AC. Therefore, the cross-correlation analysis between AB–AC does not make sense because it would be the equivalent of a comparison of A with itself. This would raise the value of the cross-correlation artificially. So, without the redundant pairs, 15 cross-correlation analyses were performed.

In terms of meaning of the CWT-F, this variable estimates the nature of the interaction between participants. A high correlation means that the participants react to a similar event in the same time but, this variable does not reveal if they have reacted in the same direction. One can perceive a raised emotional state at a particular time in reaction to an event on the video while the other one reacts to the same event but feels that the emotional state has fallen. The variable able to capture these emotional state properties is the RP (see an illustration in **Figure 5C**). The procedure to compare the participants, pair by pair using the RP variable, is the same as that used for the CWT-F variable. Therefore 10 comparisons for men and 10 comparisons for women were carried out. A Chi-square analysis to compare the distribution of the relative phase (DRP) between male and female was also carried out.

The mean of correlation coefficients obtained, for the male comparisons on the CWT-F variable, was 0.87 (±0.19 *p* < .05). The mean of correlation coefficients obtained, for the female comparison on the WT-F variable, was 0.86 (±0.18 *p* < .05). Male and female illustrated a high correlation, which implied that they both perceived events similarly. The comparison of all male results with all female results revealed a correlation coefficient of 0.98 (*p* < .05). This confirmed that both male and female participants were reacting at the same time while watching the video. In other words, similar events trigger the same response in both male and female participants.

The previous analyses and interpretations illustrate the emotion perceived by the participants from moment to moment while watching the video. This is a global picture of their emotions during the full duration of the video (119 sec). A question is raised here. Are the emotions being experienced by the participants similarly all the way through the video? Is there a gender difference if we are looking at a particular time in the CWT-F variable? The CWT method analyses the time-series so it is worth using this method to check potential differences occurring in time. The following analysis breaks down the video in five sections. For each of the sections, we applied the same statistics used in the previous paragraph (see results in **Table 2**).

**Table 2:** Correlation coefficients for the CWT-F variables for both male and female Participants.

	Section 1	Section 2	Section 3	Section 4	Section 5
Male	0.64 ± 0.12	0.74 ± 0.17	0.92 ± 0.11	0.76 ± 0.22	0.82 ± 0.18
Female	0.67 ± 0.15	0.86 ± 0.18	0.93 ± 0.08	0.74 ± 0.25	0.70 ± 0.23
Male vs. Female	0.92	0.93	0.99	0.93	0.95

The consistency observed between male and female participants in the previous analyses is confirmed here for each of the five sections. Section “WHY Does it Seem Important to Use the Cross-Wavelet Transform Method?” had the highest correlation coefficients for both male and female. We looked back at the video to see if there were specific comments or behavior from the character, which induced a stronger and even more consistent result across participants. This observation revealed that during Section “WHY Does it Seem Important to Use the Cross-Wavelet Transform Method?,” the character is talking about his feelings about someone else whereas, in the four other sections, he is mainly talking about his personal feelings of the experience during the survival tasks. At this stage and in the context of this article, it would be inappropriate to make further interpretation. However, this suggests that deeper analysis could be done, investigating specifically the raters’ judgment during a certain period of time defined by the researchers in advance.

**FIGURE 6:**
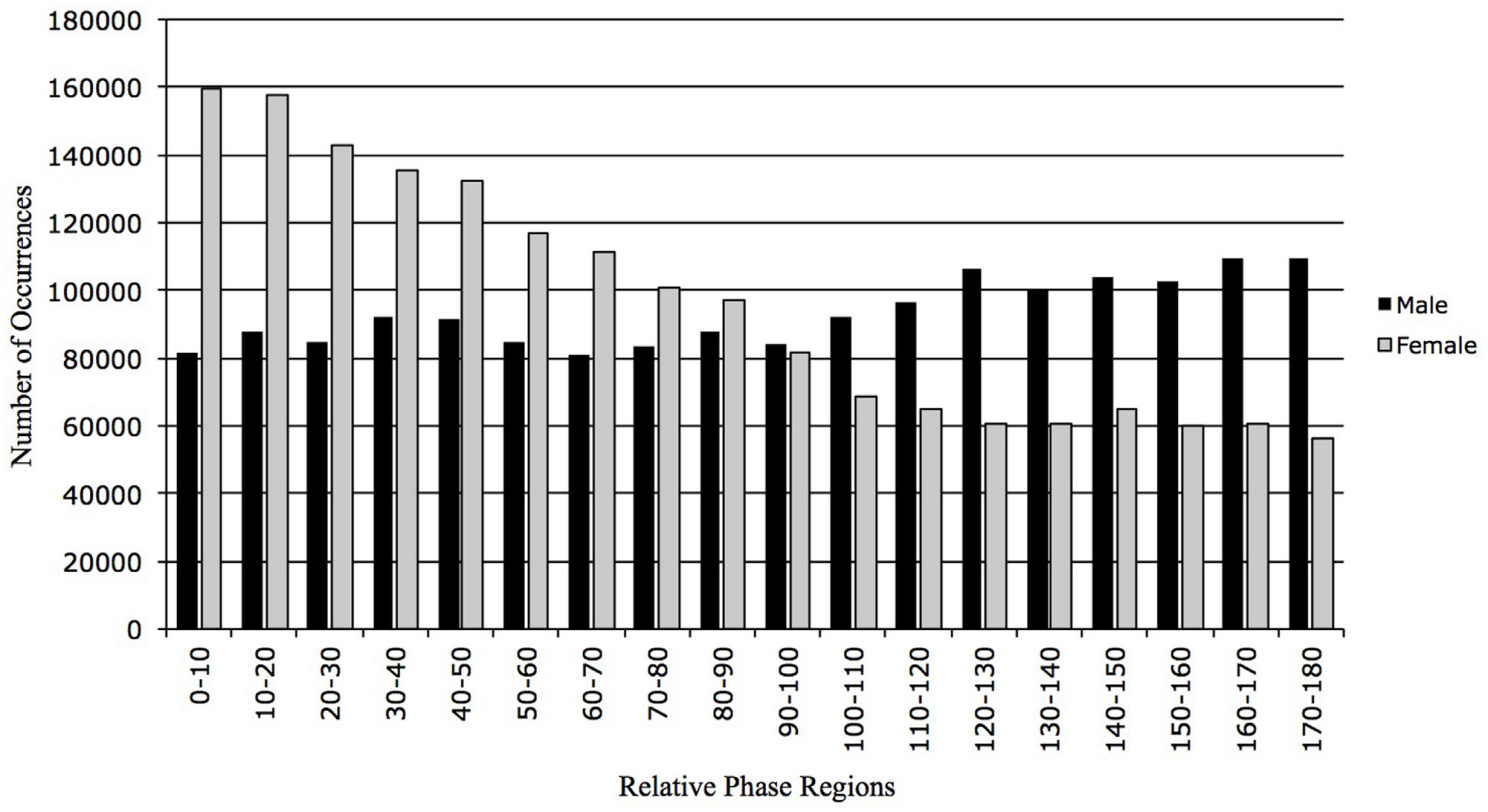
**Percentage distribution of number relative phase (NRP) occurrences for male and female.** NRP is distributed in eighteen 10° regions from 0 to 180°. The female distribution is orientated toward 0° of RP whereas the male distribution is more evenly spread over the 18 regions of RP.

The previous results illustrate that the raters react at the same time to an event but so far nothing is known about whether the participants have reacted in the same direction to the same event. The results of the RP will provide such an answer. The RP results are expressed in a range between 0 and 180°. Zero degrees correspond to the evolution of the two times-series in the same direction. If two raters have an opposite perception of the character’s emotional state, the associated behavior will correspond to 180° of RP. For the analysis, 18 regions of RP have been defined. Each region corresponds to a range of 10°. The cross-correlation analyses were conducted for the 10 pairs. As explained before, without the redundant pairs, 15 cross-correlation analyses were performed. The male correlation coefficient is 0.19 (±0.47 *p* < .05). This low correlation coefficient illustrates that there is great variability in the way they perceive the character’s emotional state. The direction of their judgment is evenly spread all the way through a range of RPs (**Figure 6**). The correlation coefficient for the female participants was 0.70 (±0.18 *p* < .05). There is more consistency between the female participants than the male participants. As we can see there is a tendency toward the 0° of RP. This suggests that the females are reacting in the same direction to the same event. They mainly perceive the same emotions while watching the video clip. The results of the standard deviation reinforce this interpretation. The female standard deviation (±0.18) is almost three times lower than the male one (±0.47). This is confirmed by a Chi-square analysis on the RP distribution, indicating a significant difference between female and male (*X*^2^ = 366264.9, df = 17, *p* < .0001).

An analysis of the RP section by section was carried out (**Table 3**). The same five sections as the ones described earlier for the CWT-F variable were identified in order to discuss the results altogether.

**Table 3:** Correlation coefficients for the relative phase for both male and female participants.

	Section 1	Section 2	Section 3	Section 4	Section 5
Male	0.08 ± 0.44	0.01 ± 0.39	0.25 ± 0.41	0.00 ± 0.44	0.08 ± 0.48
Female	0.77 ± 0.24	0.63 ± 0.24	0.83 ± 0.51	0.72 ± 0.45	0.69 ± 0.53

The RP analysis section by section revealed similar results to the RP analysis of the full video. The male raters illustrated a low consistency while the female participants revealed a relatively high correlation for each of the sections and particularly at Section “WHY Does it Seem Important to Use the Cross-Wavelet Transform Method?” The statistical analysis of the RP distribution is significant for each of the five sections (Section 1: *X*^2^ = 71664.6, df = 17, *p* < .0001; Section 2: *X*^2^ = 169759.9, df = 17, *p* < .0001; Section 3: *X*^2^ = 153759.2, df = 17, *p* < .0001; Section 4: *X*^2^ = 164405.6, df = 17, *p* < .0001; Section 5: *X*^2^ = 15966.8, df = 17, *p* < .0001). These statistical differences in the DRP confirm the gender differences described above with the correlation coefficients. Another interesting result concerns Section “WHY Does it Seem Important to Use the Cross-Wavelet Transform Method?” This section has the highest correlation coefficients for both male and female raters. This result is in line with the results observed for the CWT-F variable. It would be inappropriate to interpret these results any further, but they suggest that specific triggers may provoke a convergence of the perception of emotional state.

Globally, these results suggest that both male and female raters seem to react consistently to the different events occurring in the video. Changes in emotional state seem to be perceived at the same time across the two genders. Interestingly, the only variable illustrating a gender differences is the RP. The RP expresses the direction of the emotional state perceived by the raters. These differences in gender suggest that the male raters perceive the changes in the emotional state in different ways whereas the female participants react in phase to the event occurring in the video.

### CONCLUSION

Throughout this tutorial, we aimed to shed light on how the CWT method can be a useful tool in analyzing the interaction between times-series. The CWT method allows us to depict a continuous RP for the whole range of frequencies present in time-series and in doing so overcomes previous limitations present in other methods. Time-series analysis through the “local” and “multi-scale” properties of the CWT method permits us to investigate any complex synchronization between time-series in numerous different types of areas in psychology.

## Conflict of Interest Statement

The authors declare that the research was conducted in the absence of any commercial or financial relationships that could be construed as a potential conflict of interest.

## Footnotes

1 The term wavelet means a small wave.

2 Using a ‘mouse-paradigm’ which consisted in moving a computer mouse to track participants moment-to-moment self-evaluation based on their own recorded narrative.

3 For more detailed explanations about the cross-spectral methods please refers to Boker et al. (2002) and Issartel et al. (2006).

4 See http://emotion-research.net/projects/humaine for a detailed explanation of this European Network of Excellence. From the website the readers can have access to the data used in this section of the article.

5 A customized program made by two co-authors (Philippe Gaillot and Thomas Bardainne) in the software language C++ transformed raw data into WT and CWT (see hyperlink in the text to download the “Cook-book” and the source code). The WT and CWT were analyzed using Matlab software (MATLAB R2013b, MathWorks Inc., Natick, MA, USA). Note that on the web different codes and method descriptions are available and ready to be used.
